# Macroscopic Examination of Multiple-Shot Cattle Heads—An Animal Welfare Due Diligence Tool for Abattoirs Using Penetrating Captive Bolt Devices?

**DOI:** 10.3390/ani9060328

**Published:** 2019-06-06

**Authors:** Andrew Grist, Toby G. Knowles, Stephen Wotton

**Affiliations:** Bristol Veterinary School, University of Bristol, Langford House, Langford, Bristol BS40 5DU, UK; toby.knowles@bristol.ac.uk (T.G.K.); steve.wotton@bristol.ac.uk (S.W.)

**Keywords:** animal welfare, abattoir, Animal Welfare Officer, captive bolt, cattle, due diligence, multiple stun attempts

## Abstract

**Simple Summary:**

The most common method of stunning cattle prior to bleeding in an abattoir is a penetrating captive bolt device, which is basically a piston driven forward by expanding gas produced by a blank cartridge. This creates a concussed state in the animal on impact with the skull, followed by penetration to prevent recovery by destroying key parts of the brain. The successful application of this device requires the correct cartridge strength for the species and accurate placement of the device. This paper examines the heads of animals that have received multiple shots in an attempt to ascertain the cause, to provide abattoirs with training material and an investigative tool to reduce the occurrences of multiple shots and reduce the consequent negative effects on animal welfare at slaughter.

**Abstract:**

Ideally, the application of a penetrating captive bolt device to render cattle immediately unconscious prior to slaughter would be 100% effective. Unfortunately, due to various factors this is not always the case. This paper examined, as an initial proof of concept, 12 bovine heads which had received more than one shot from a penetrating captive bolt, collected from various abattoirs within the United Kingdom. The heads were frozen to facilitate splitting on the medial plane to prevent distortion of soft tissue and each sagittal section was examined macroscopically to ascertain if this method could be used to determine the reasons for repeated stun attempts. In 10 out of 12 heads, shot placement was the determining factor, in one other head it was felt that anatomical variation was the reason and the twelfth head demonstrated signs of gun malfunction as the likely cause. This work provides evidence for a larger trial to facilitate the production of guidance for the abattoir industry, the Animal Welfare Officer and regulators on the examination of heads as part of an investigation of failures of a mechanical stunning system and to provide training material for slaughter staff tasked with effectively stunning cattle.

## 1. Introduction

Unless undertaking religious slaughter practice, pre-slaughter stunning of cattle is a legal requirement of both European legislation [1] and United Kingdom National Legislation [2,3,4]. In addition, the European Regulations include the requirement for the presence of an Animal Welfare Officer in each facility slaughtering more than 1000 livestock units per annum, each bovine being 1 livestock unit [1].

The majority of bovines in the United Kingdom are stunned mechanically using penetrating captive bolt devices [5], with the exception of (a) abattoirs using systems such as the Jarvis electric system to stun-kill cattle, (b) those opting for non-stun slaughter under derogation of slaughter according to religious rite (Article 4) [1], and (c) those opting for the use of free bullets. The object of mechanical stunning is to produce a state of unconsciousness without pain which continues until cortical brain death through exsanguination or pithing [2,3,4].

**Figure 1:**
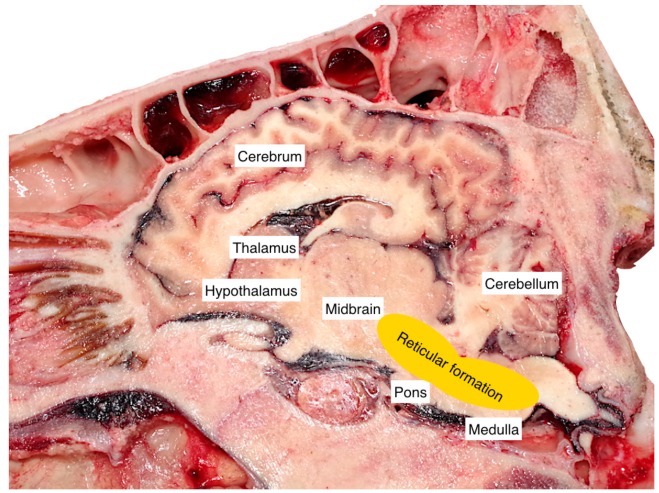
Position of reticular formation—right sagittal section of bovine head (adapted from Terlouw et al. [7]).

The use of a penetrating captive bolt has been recognised as a two-stage process: a concussed state following the transfer of kinetic energy from the bolt to the cranium followed by the effect of penetration and subsequent withdrawal of the bolt, which can prevent recovery from the concussed state [6]. The shot is targeted to affect either the cerebral hemispheres on a large scale, the reticular formation, ascending reticular activating system or the median thalamus bilaterally [7] (Figure 1). The European Food Safety Authority [8] state that the extent of the damage determines the outcome of the application, and this is dependent on the size and shape of the head, skull structure and thickness, density and porosity of the bone at the recommended shooting site and on the equipment used.

It should be possible and desirable, to successfully stun 100% of the animals in a commercial abattoir [9,10]. Occasionally, due to various factors such as anatomical differences, gun maintenance and failure, cartridge strength [11] and condition, shot position and access to the animal, multiple shots have to be administered to an animal to ensure an effective stun [6,10,12,13,14]. Gregory et al. [15] showed that a higher percentage of bulls (16%) demonstrated inadequate stunning compared with female cattle (6%). Atkinson et al. [14] reviewed other papers and gave a range of between 9% and 32% of bovines that were not stunned at the first attempt [12,15]. Fries et al. [16] examined 8879 cattle skulls at 2 head deboning plants, focusing on the precision of shot placement, finding that 4.0% and 3.1% of heads examined in the two plants had poor placement and that 284 (3.2%) of the total heads presented with two shot holes and 4 (0.05%) presented with 3 holes. These authors stated that ‘measurements of the distance from the ‘ideal’ position and the penetration angle of the bolt do not reflect the efficacy of stunning.’ However, their conclusion was based on post mortem examination rather than observation of the animal at the point of stun and pointed out that secondary shots may be confirmatory or due to uncertainty. However, the authors did raise the point that the position and direction of the shot may serve as an indirect control or assessment point [16].

European Union and UK domestic legislation [1,2,3,4] require some form of head restraint to permit accurate stunning of an animal, however, designs vary. Von Wenzlawowicz et al. [13] reported that 35% of cattle were inaccurately shot when abattoirs were not equipped with restraint devices to steady the head prior to the application of the captive bolt gun.

Council Regulation (EC) 1099/2009 on the protection of animals at the time of killing [1] requires monitoring procedures to be put in place (Article 16) [1] and also that methods should be reviewed in case of failure (Article 16) [1]. This paper introduces the concept of macroscopic examination of the sectioned heads of bovines receiving multiple applications as an additional tool that the Animal Welfare Officer or government enforcement official could use in these cases to attempt to ascertain likely reasons for these failures; in conjunction with other investigations that should be undertaken in an effort to understand and reduce the occurrences of multiple stun attempts within abattoirs. This pilot study was designed to examine the value of macroscopic examination of heads for shot position variability, anatomical variation and other factors that may contribute to the need for secondary and tertiary stun attempts as a guide for these investigations. Finnie, [17] demonstrated that penetrative captive bolt use produces a well demarcated haemorrhagic tract within the brain structure, which can be used in conjunction with exterior shot position to estimate the outcome and affected cerebral structures from each shot.

## 2. Materials and Methods

Twelve flayed heads from animals that incidentally received multiple shots during the course of production were sourced from five cattle abattoirs within the United Kingdom together with, when available, details of the device used, the cartridge strength, a record of the number of shots, the kill number for the day, the breed, sex and age of the animal. The animals were assessed by licenced slaughter staff during the course of their daily operation as being ineffectively stunned based on the standard accepted behavioural indicators of continued brain function used in the abattoir setting (rhythmic breathing and/or corneal reflex and/or eyeball rotation [8,18]) and were subsequently reshot until effective stunning resulted. These heads were then sent to the authors for examination. The heads were hard frozen to facilitate sectioning without lesion distortion, with an electric band saw (Startrite, Meatmaster, UK) along the sagittal plane. The cut surface was gently washed with water at room temperature from a long-nosed polyethylene wash bottle (Fisher Scientific, Leicestershire, UK) to remove bone dust. The brain and cranial cavity were examined macroscopically to ascertain shot position and wound tract, to deduce shot order. The assumption was made that the ‘on target’ shot tract was the terminal shot position, all others were denoted numerically or alphabetically starting at the rostral position for descriptive purposes. Heads were photographed with a Nikon D5100 digital camera (Nikon Corporation, Tokyo, Japan). Shot holes and trajectories were assessed using an 8 mm diameter stainless steel trocar (Surgical Holdings UK, Essex, UK) to replicate the path of the captive bolt (nominal average bolt diameter = 11.9 mm). Frontal sinus thickness was measured at the point of the recommended shot position with a standard photographic ruler (Forensics Source ABFO No. 2 Sign Photo Ruler, Amazon UK). The details of kill number, age, gender, breed and frontal sinus thickness were recorded so that these variables can be included in possible future larger scale studies as possible factors in the requirement for repeated shot application.

## 3. Results

The findings from the post mortem inspections of the heads are detailed in Table 1, with descriptions of the findings for three of the heads, Head 8 (multiple shots due to positioning issues), Head 4 (multiple shots possibly due to anatomical variation) and Head 12 (multiple shots possible due to gun performance) reported below.

**Figure 2:**
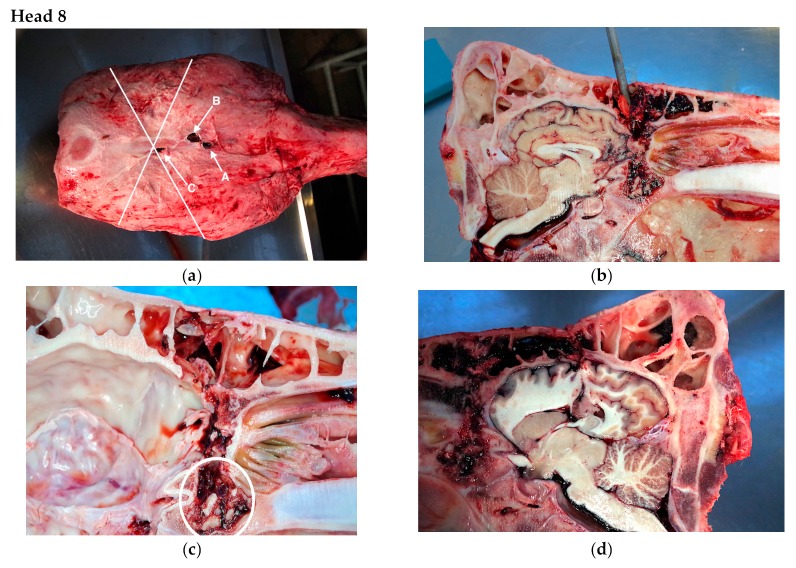
Kill 108 of day. British Blue Cross Male Aged 15 months 27 days—3 Shots (**a**) Head 8 shot placement nominal numbering for descriptive purposes. Crossed lines demonstrating ‘ideal’ shot position. (**b**) Left sagittal section. Shot A and B, trocar demonstrating tracts. (**c**) Left sagittal section, brain removed. Shot A and B, demonstrating wound tracts and bone shards (circled). (**d**) Right sagittal section demonstrating haemorrhagic tract left by penetrating captive bolt.

The animal was reportedly shot three times (Figure 2a). On the split head, the bolt tract of shots A and B were difficult to differentiate, however both shots were positioned lower than the ideal position (Figure 2b) and followed a path that did not enter the cerebral cortex. The tract of the bolt entered through the frontal sinus, skimmed the frontal cerebrum and after passing through the sphenoidal sinus terminated in the anterior sphenoid bone. (Figure 2c). Bone shards propelled by the bolt were found in the ventral sphenoidal sinus. Shot C was positioned almost exactly at the ‘ideal’ shot position, at which point the sinus thickness was approximately 26 mm. A second 1 cm cut was made through the right sagittal section. The bolt tract passed through the sinuses below the frontal bone and through the medial dorsal cerebrum. (Figure 2d). Petechial haemorrhages were evident within the pons and medulla. There were no abnormalities found within the cranium that would suggest other than initial shot placement as the reason for the further captive-bolt application.

**Figure 3:**
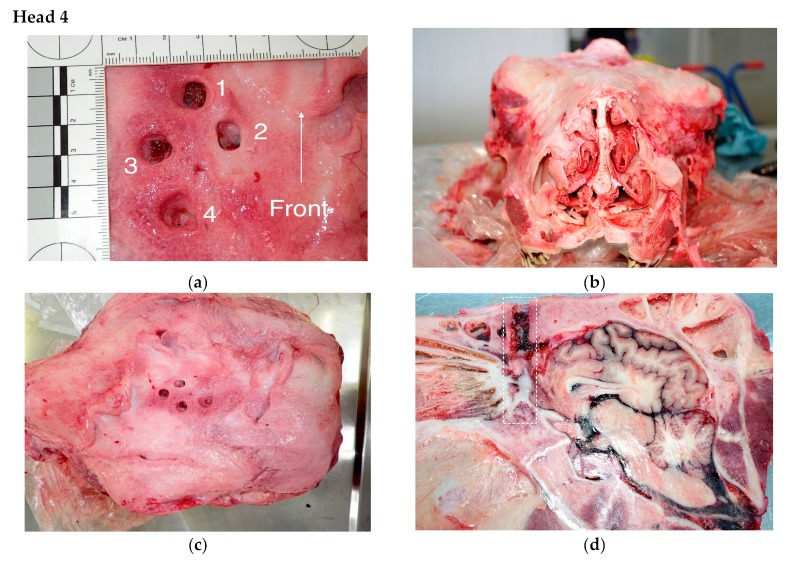

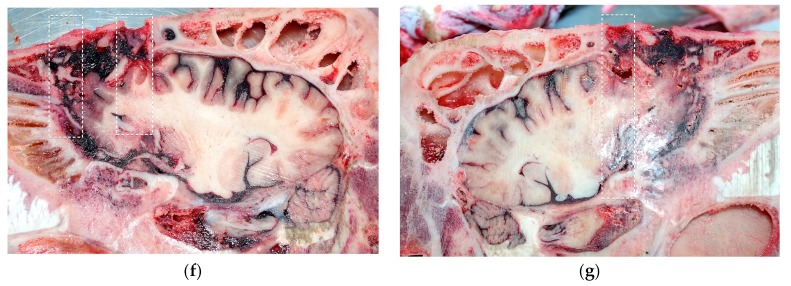
Kill 111 of day. Aberdeen angus cross bull Aged 12 months 19 days—4 Shots (**a**)Head 4 shot placement nominal numbering for descriptive purposes. (**b**) Head 4. Assymetry of head due to anatomical deformity. (**c**) Fibrous material covering the cranium, up to 5mm thick in areas. (**d**) Right sagittal section. Shot 1 pathway denoted by dotted lines. (**f**) Right sagittal section Shot positions 2 and 3 pathways denoted by dotted lines. (**g**) Left sagittal section. Shot 4 pathway denoted by dotted lines.

The animal had been shot four times (Figure 3a). The unusual 5mm thick covering of fibrous material over the cranial vault may have absorbed some of the impact kinetic energy reducing the effectiveness of the captive bolt device (Figure 3c). In addition, there were slight anatomical abnormalities within the skull structure that may have reduced the effectiveness and accuracy of positioning (Figure 3b). Based on the standard model of shooting at the intersection of two imaginary lines drawn from the top of the eyes to the base of the opposite horn bud, at least two of the shots may have been more cranial than the ideal position, this may have been exacerbated by the slight elongation of the brain within the cranium. Using the nominal shot sequence based on position only: shot 1 barely grazed the fontal cerebrum and entered the dorsal sinuses and in this case based on the sagittal section, was approximately 5 cm lower than a position that would penetrate the mid brain (Figure 3d,e). A similar effect was seen with shot 2. Shot 3 was placed approximately 2 cm caudal to shot 2 but passed through the frontal cerebrum (Figure 3e). Shot 4 was higher on the head and angled more toward the midbrain but due to the slight anatomical differences did not reach the reticular system (Figure 3f). In this case it was concluded that the first shots were placed lower than the ideal position, but that a thick subcutaneous fibrous tissue layer, combined with anatomical variation from the norm in both the skeletal structure and brain anatomy was the cause of the requirement for multiple shots. Each shot was far enough away from previous shot positions for impact on solid bone.

**Figure 4:**
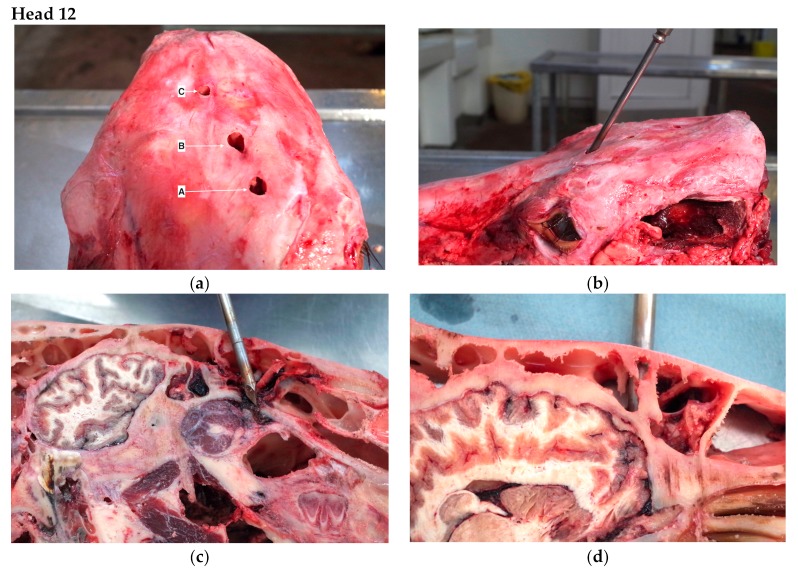

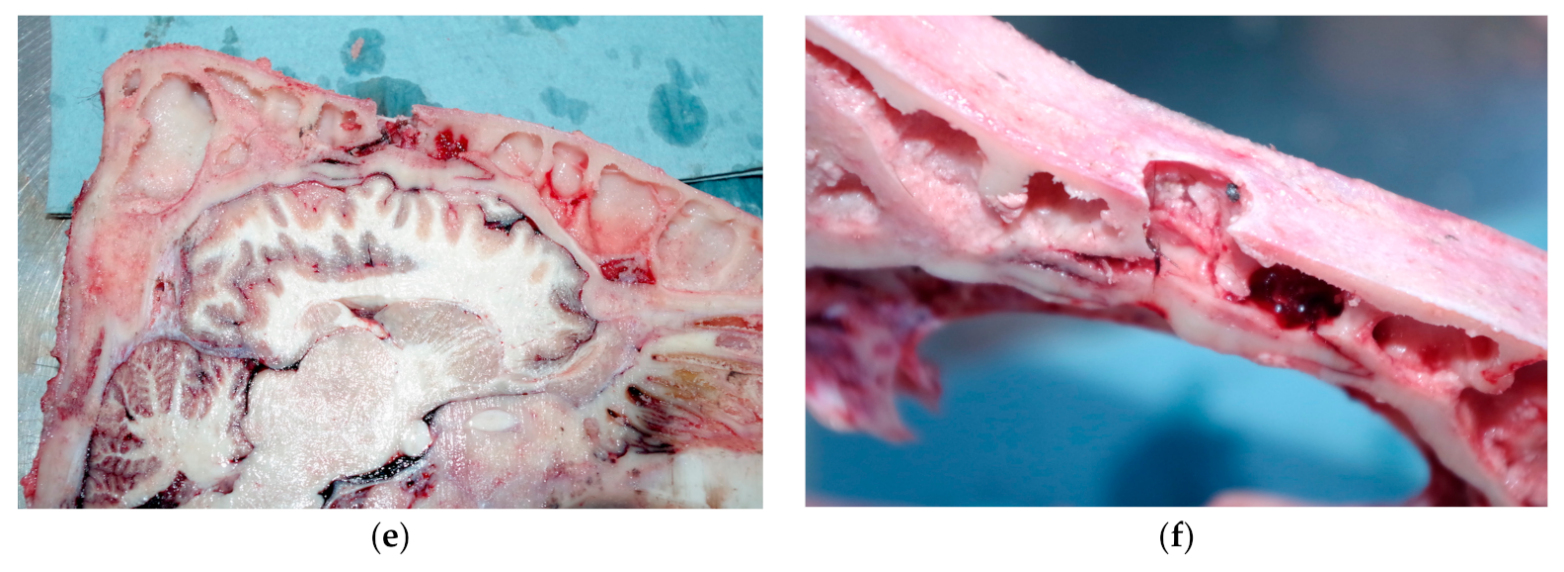
Kill 106 of day. Limousin Cross Heifer Aged 22 months 12 days—3 Shots (**a**) Shot numbering for descriptive purposes. Shot A denoting most rostral position and C the most caudal placement of shots. (**b**) Angle of shot A replicated by 8 mm trocar. (**c**) Inferred Shot A position at the level of the frontal sinus and passed into the posterior middle nasal concha. (Left sagittal section). (**d**) Shot position B Left sagittal section. (**e**) Shot C trajectory (Left sagittal section). (**f**) Shot C—No corresponding hole in lower frontal sinus (Left sagittal section).

Two of the shots were positioned below the ‘ideal’ shot position (described above). The shot order lettering is purely for descriptive purposes and does not indicate an assessment of shot order (Figure 4a). Shot A was angled at 50° to the perpendicular and approximately 4 cm to the left of the midline and entered the nasal sinus (Figure 4b,c). Shot B was perpendicular to the head but below the ‘ideal’ position, entering the cranial vault passing through the anterior frontal cerebrum and the sphenoid sinuses and terminating in the frontal sphenoid bone (Figure 4d). Shot C was unusual in that it was in a position that would be considered appropriate (Figure 4e) but did not extend more than 2 cm into the frontal sinus (Figure 4f) and did not enter the cranial vault, suggesting either a weak cartridge or the device being held at least 12.5 cm from the animals’ head when fired (based on the sinus thickness being 22 mm at this point and the quoted extension of the bolt being 145 mm with no recuperator sleeves).

The abattoir reported that the animal went down and was released from the pen before attempting to regain posture before it was re-stunned ‘freestanding’. It would appear that either shot A or C were applied when the animal was restrained in the stun box. If it was shot A this is a positioning issue; however, if it is shot C it may be a failure of contact or cartridge.

## 4. Discussion

This pilot study suggests that the macroscopic examination of bovine heads that receive more than one shot can provide allow reasonable conclusions to be drawn as to possible causes as part of the investigation into the need for extra shot application. In the small sample of 12 heads examined, shot position accounted for 83.33% of the repeated shots, one head presented anatomical variation (a thick fibrous layer over the cranial vault and a differing skull and brain morphology) that could account for the required extra stun attempts and one head provided evidence that one of the shots either did not have enough power due to cartridge strength or the gun was held over 10 cm from the head when the shot was applied, which, given the legislative requirement for head restraint [1,2,3,4], would be an unlikely scenario. Examination of the external head after flaying (for facilitating post mortem inspection) [20] to ascertain if the animal was stunned is a flawed procedure, the assessment of a successful stun being based on behavioural indictors at the point of stunning and cannot be a retrospective act. A shot position away from the recommended position does not unequivocally relate to a poor stun and vice versa. However, if the guns are numbered and rotated so they get equal use, second stun requirements from the same gun number can be an indication of mechanical issues with that tool, so should be recorded and actioned.

The UK legislation requires that no person may use a penetrative captive bolt device to stun an animal unless …’the device is positioned and applied so as to ensure that the projectile enters the cerebral cortex’ [2,3,4]. Terlouw et al. [7] reviews our understanding of the factors that produce a stunned state which indicate that the target should be the reticular activating system and/or the ascending reticular activating system and/or the median thalamus bilaterally and/or affecting the cerebral hemispheres on a massive scale, rather than just the cortex. Several of the heads examined for this project had initial shots that entered the cerebral cortex, but in a position and angle too frontal to affect the mid brain and brain stem. The findings also support the findings of Gilliam et al. [21] who suggest a higher optimal shot location to produce maximum brain stem damage and these authors would suggest that with older animals and continental breeds, especially males, that this higher position is adopted in conjunction with the highest available grain cartridge.

**Figure 5:**
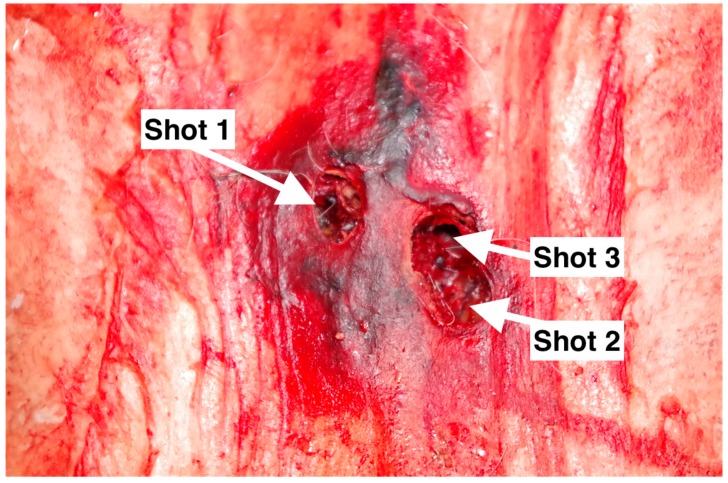
Figure-eight elliptical entrance wound created by shots 2 and 3 of head number 3.

The post mortem findings in cases where shots were applied adjacent to each other, producing an elliptical or ‘figure-eight’ entrance hole (Figure 5) followed by a third shot supports the findings of Daly and Whittington [22] that the transfer of kinetic energy to an intact skull is a key requirement for a successful stun.

As a pilot study, the sample size was too small to examine the effect of kill number, gender, breed or age on the efficacy of stunning but in terms of anatomical variation, we must include consideration of animal gender, Atkinson et al. [14] reported that bulls were three times as likely to receive an inadequate stun than the other cattle classes and this corresponded to the findings of Gregory et al. [15]. In this study 7 of the 9 animals we received data for were male, and only 4 out of 12 animals were over 30 months of age.

The sanctioning of staff who apply more than one shot simplifies a complex issue and cannot be recommended as a primary response. The welfare of the animal is an obvious priority, and repeated stun attempts are indicative of a failure of the system used and must be remedied by improvement in the system rather than by sanction; the current European legislation [1] requires monitoring of stunning and that review should be undertaken in the case of failure (Article 16) [1]. The fact that the slaughter operative recognises the requirement for, and applies, a second or further stun based on behavioural indicators or a precautionary measure should, in the authors’ view be applauded and should be undertaken without hesitation due to fear of sanction, which was raised by Fries et al. [16] who questioned if some operators may avoid further shots to reduce attention to their own performance, and that the operator must be encouraged to shoot again if in doubt.

## 5. Conclusions

The macroscopic examination of sectioned bovine heads, in conjunction with other investigations, can be used as an aide to assess the reasons for an ineffective stun.

If the abattoir is equipped with a freezer that would accommodate a bovine head, and access to a bandsaw, with a trained operative following health and safety procedures, it is possible to assess the probable causes for repeated stun attempts. In the European Union the bone dust and washings from the band saw would have to be treated as Specified Risk Material (SRM) [23] but this should not pose an issue as abattoirs processing adult bovines will have suitable systems in place for the storage, staining and disposal of SRM produced, as part of their standard operating conditions.

Assessment of macroscopic lesions in bovine heads can also provide training material for slaughter staff should positioning of the shot be found to be an issue, and this will improve the welfare of other animals processed by the operatives.

## Figures and Tables

**Table 1:** Information for heads examined by post mortem inspection.

No.	Kill Number	Age				Number of Shots	Frontal Sinus Thickness	Suggested Reason for Multiple Shots Following Examination
		Months	Days	Sex	Breed ^1^			
1	119	15	20	-	-	4	22 mm	Position
2	112	24	10	-	-	4	27 mm	Position
3	161	27	2	M	CHX	3	26 mm	Position
4	111	12	19	-	AAX	4	25 mm	Anatomical
5	48	15	6	M	LIMX	4	20 mm	Position
6	171	82	27	F	SIMX	4	18 mm	Position
7	190	137	37	M	AA	5	30 mm	Position
8	108	15	27	M	BRBX	3	26 mm	Position
9	82	14	17	M	CHX	5	30 mm	Position
10	117	69	17	M	ST	4	13 mm	Position
11	185	118	20	M	AA	3	30 mm	Position
12	106	22	12	F	LIMX	3	22 mm	Gun performance

^1^ Breed codes according to the British Cattle Movement Service Official cattle breeds and codes [19]. The sinus thickness is given at the midline at the ‘ideal’ shot position.
